# Minimally invasive tooth extraction: the science and clinical strategies of socket preservation: a comprehensive review

**DOI:** 10.3389/froh.2026.1834419

**Published:** 2026-08-12

**Authors:** Patr Pujarern, Wiphu Srisawan, Boontharika Chuenjitkuntaworn, Sasipa Thiradilok, Dinesh Rokaya, Suphachai Suphangul

**Affiliations:** 1 Department of Advanced General Dentistry, Faculty of Dentistry, Mahidol University, Bangkok, Thailand; 2 Clinical Sciences Department, College of Dentistry, Ajman University, Ajman, United Arab Emirates; 3 Center of Medical and Bio-Allied Health Sciences Research, Ajman University, Ajman, United Arab Emirates

**Keywords:** atraumatic extraction, immediate implant placement, minimally invasive extraction, socket preservation, tooth extraction

## Abstract

**Introduction:**

Tooth extraction inevitably initiates alveolar socket remodeling and resorption, particularly of the buccal bone, which may compromise future implant placement and esthetic outcomes.

**Methods:**

This narrative review synthesized current evidence on post-extraction socket healing, alveolar ridge preservation, and minimally invasive tooth extraction techniques aimed at reducing surgical trauma and maintaining hard and soft tissue integrity.

**Results:**

The literature demonstrates that significant dimensional changes occur following tooth extraction, with the greatest bone loss occurring in the buccal aspect during the early healing phase. Minimally invasive approaches, including periotomes, piezotomes, magnetic mallets, sonic instruments, physics forceps, vertical extraction systems, vestibular root extraction, enzymatically assisted extraction, socket-shield techniques, and root sectioning methods, have shown varying degrees of effectiveness in preserving alveolar bone, reducing postoperative pain and complications, and facilitating immediate implant placement. Adjunctive socket preservation strategies, such as alveolar ridge preservation and soft tissue grafting, further enhance maintenance of ridge dimensions and esthetic outcomes.

**Conclusion:**

Atraumatic extraction is a critical component of socket preservation and successful implant therapy. While several minimally invasive techniques have demonstrated promising clinical benefits in reducing tissue trauma and preserving alveolar architecture, the quality and quantity of evidence vary among techniques. Further well-designed clinical studies are required to establish standardized protocols and strengthen evidence-based clinical decision-making for minimally invasive extraction and socket preservation.

## 1 Introduction

Tooth extraction is one of the most basic routine dental treatments performed by dental practitioners for various reasons, including caries and periodontitis, endodontic problems, orthodontic considerations, failure of eruption, dental trauma, esthetics, part of a prosthetic plan, and other medical reasons (1, 2). Often, tooth extraction requires the separation of dental roots, followed by the use of elevators and forceps with the principles of wedge, lever, wheel, and axle, or a combination of these (3, 4). The principles of exodontia involve simple or basic exodontia that uses luxation, bone expansion, and forceps delivery, and complex exodontia with techniques other than simple exodontia in conditions such as extensive carious lesions, teeth with internal resorption, endodontically treated teeth, hypercementosis, abnormal root morphology, difficult anatomic locations, ankylosed teeth, teeth that will be used for autotransplantation, and multi-rooted teeth located in areas where bone preservation is critical for implant placement, as indicated by surgical extraction (5–7). Surgical extraction usually follows the following steps: elevation of a mucoperiosteal flap, ostectomy, sectioning of the tooth, luxation and removal of the roots, removal of periapical lesions if present, debridement of the surgical field, the elimination of sharp bone edges, and wound closure (5, 8).

Conventional tooth extraction techniques primarily involve the use of instruments, elevators, and forceps to luxate the tooth, continue the process of socket bone expansion, and disrupt the periodontal ligament (PDL) attachment. The mechanical principles and simple machines required in the process are a lever, wedge, wheel, and axle. A dental elevator consists of a handle, shank, and blade. A larger handle diameter allows it to be held and grasped in the palm, while some have a flattened area for the fingers to rest. The shank connects the blade and handle in the same line, and some shanks that align perpendicularly are available. The blade configurations can be straight, curved, triangular, or pointed to aid the user. Another main tool for extraction, the forceps, could be used in five major motions: apical motion to insert the beaks down into the PDL space and displace the center of rotation more apically, moving the fulcrum to gain mechanical advantage; buccal force to expand the buccal plate at the crest of the ridge; lingual or palatal pressure to expand the linguocrestal bone; rotational pressure to rotate and cause internal expansion of the tooth socket and tearing down the PDL, especially in single-rooted teeth with a conical shape; and tractional force to deliver the tooth from the socket after adequate bone expansion. The five forceps movements used in tooth extraction—apical pressure, buccal and lingual/palatal expansion, rotational force, and final traction—are generally applicable to both maxillary and mandibular teeth but are not always in the same sequence or with equal emphasis. Their application depends on factors such as bone density, root morphology, and tooth position. The maxillary teeth typically allow greater buccal movement and more frequent rotation, whereas the mandibular teeth require more controlled buccolingual forces and limited rotation. Thus, these motions are applied flexibly rather than in a fixed order. The points are to expand enough of the bone socket by wedging with the beaks of the forceps and ultimately remove the tooth from the socket. Mostly, tooth removal can be performed with close delivery, but in cases where inadequate surgical access cannot be achieved with these earlier techniques, open or surgical extraction is indicated to safely remove a tooth or its remaining roots (1, 9). This narrative review provides an overview of socket healing following extraction and various innovative approaches to minimally invasive extraction for socket preservation.

## 2 Anatomic and physiologic changes following tooth extraction

After tooth extraction, the alveolar ridge undergoes atrophy, the wound heals with epithelial coverage, and an edentulous ridge remains (10). The resorption of the buccal surfaces was greater than that of the palatal surfaces in the maxilla and in the same manner in the mandible, that is, greater on the buccal surfaces than on the lingual surfaces. Hard and soft tissue remodeling may result in a concave profile at the subsequent edentulous ridge (11). Pietrovski and Massler (12) found a greater amount of buccal resorption in the molar area than in the incisor and premolar regions of the maxilla and mandible. Resorption did not significantly differ between the incisor and premolar regions. Greater buccal resorption after extraction, particularly in the mandibular molar regions, is primarily attributed to the loss of bundle bone (which is highly dependent on the periodontal ligament for its blood supply) combined with higher occlusal loading and larger socket dimensions associated with multi-rooted teeth (13, 14). Although both the buccal and lingual plates may be cortical, the buccal plate tends to be more susceptible to remodeling owing to its structural and vascular characteristics, whereas the lingual plate may be relatively preserved by muscular support and denser bone. Overall, molar regions exhibit greater ridge resorption than the premolar and incisor areas because of wider alveoli, complex root morphology, and increased biomechanical forces that accelerate post-extraction remodeling (2, 15, 16).

**Figure 1:**
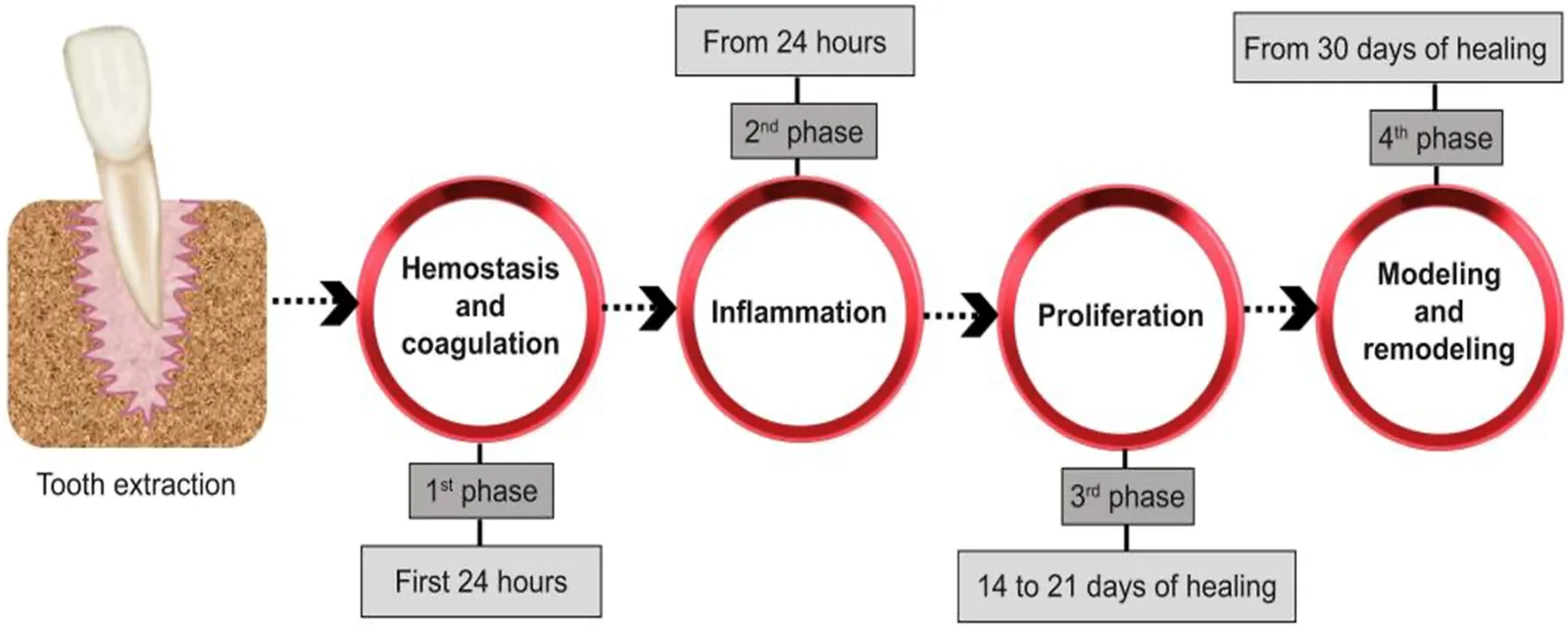
Biological events in socket healing following tooth extraction. Reproduced from “Biological events occurring in the socket after tooth extraction described into a sequence of four time-dependent phases” by Pedro de Sousa Gomes, Povilas Daugela, Lukas Poskevicius and Lorena Mariano, Maria Helena Fernandes, licensed under CC BY-NC-ND 3.0.

**Figure 2:**
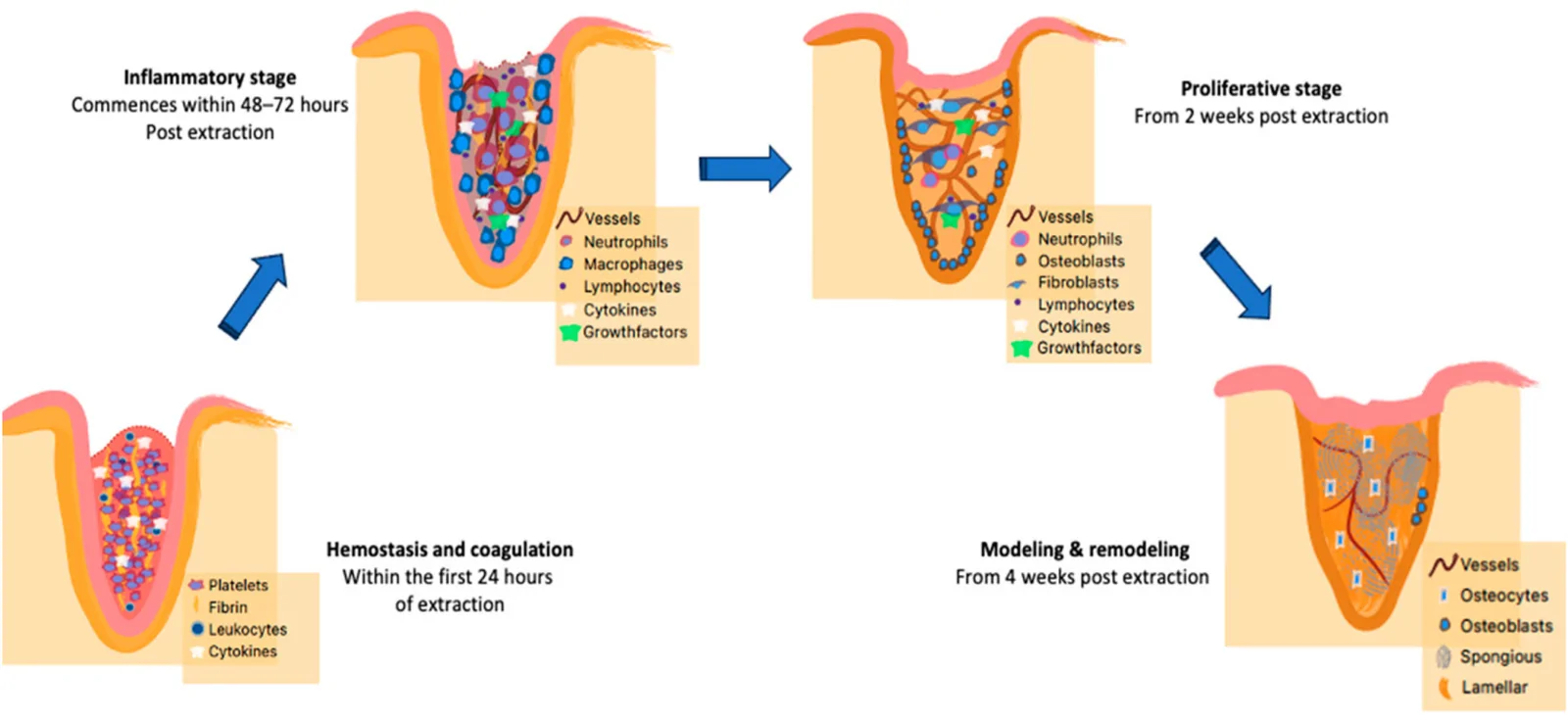
Diagram representing the time sequence of tissue regeneration in human extraction wound healing. Reproduced with permission from Reproduced from “Graphical representation of the 4 stages/phases of socket healing from the time of tooth extraction, detailing the timing of the process and the physiological events involved” by Samuel E. Udeabor, Anja Heselich, Sarah Al-Maawi, Ali F. Alqahtani, Robert Sader and Shahram Ghanaati, licensed under CC BY.

As shown in Figure 1, various biological events in socket healing following tooth extraction are time-dependent (17). The time sequence for normal human tissue regeneration is shown in Figure 2 (18). According to Amler (19), the time sequences of human tissue regeneration and wound healing are as follows: epithelial proliferation (in 4 days), beginning of bone formation (in 7 days), organization of clots (in 71–21 days), epithelial fusion (in 22 days), and bone complications (in 35 days). Furthermore, wound healing can be divided into four stages: hemostasis and coagulation, inflammation, proliferation, and remodeling (Figure 2) (18). Fibroblasts play important roles in wound healing. After hemostasis, they are involved in all stages of the healing process. Fibroblasts orchestrate the wound healing process through crosstalk with other cell types, production of growth factors, chemokines, matrix metalloproteinases (MMPs), and extracellular matrix components, and transdifferentiation into myofibroblasts, which are responsible for wound contraction (20).

**Figure 3:**
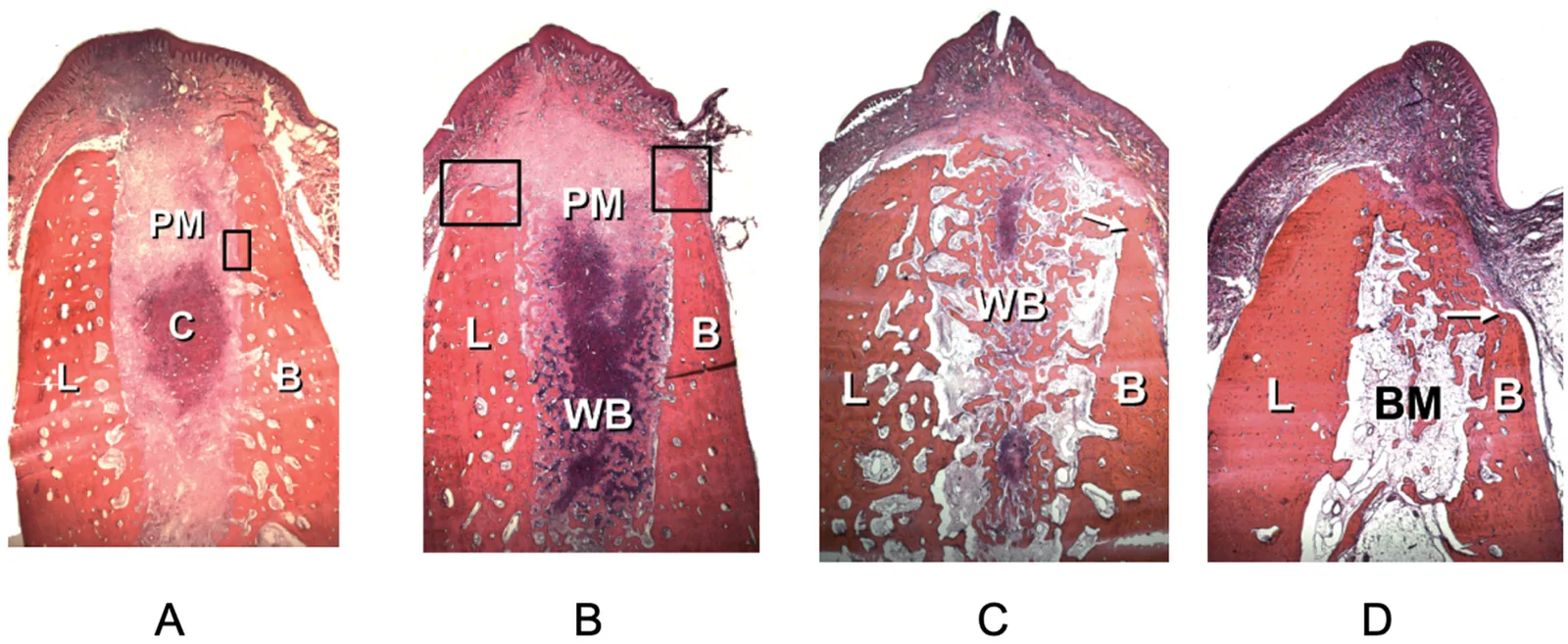
Histological examination sections above show an overview of the healing of the extraction site in **(A)** 1 week of healing, **(B)** 2 weeks of healing, **(C)** 4 weeks of healing, and **(D)** 8 weeks of healing. Reproduced with permission from John Wiley and Sons (15) - Copyright © Blackwell Munksgaard 2005.

**Figure 4:**
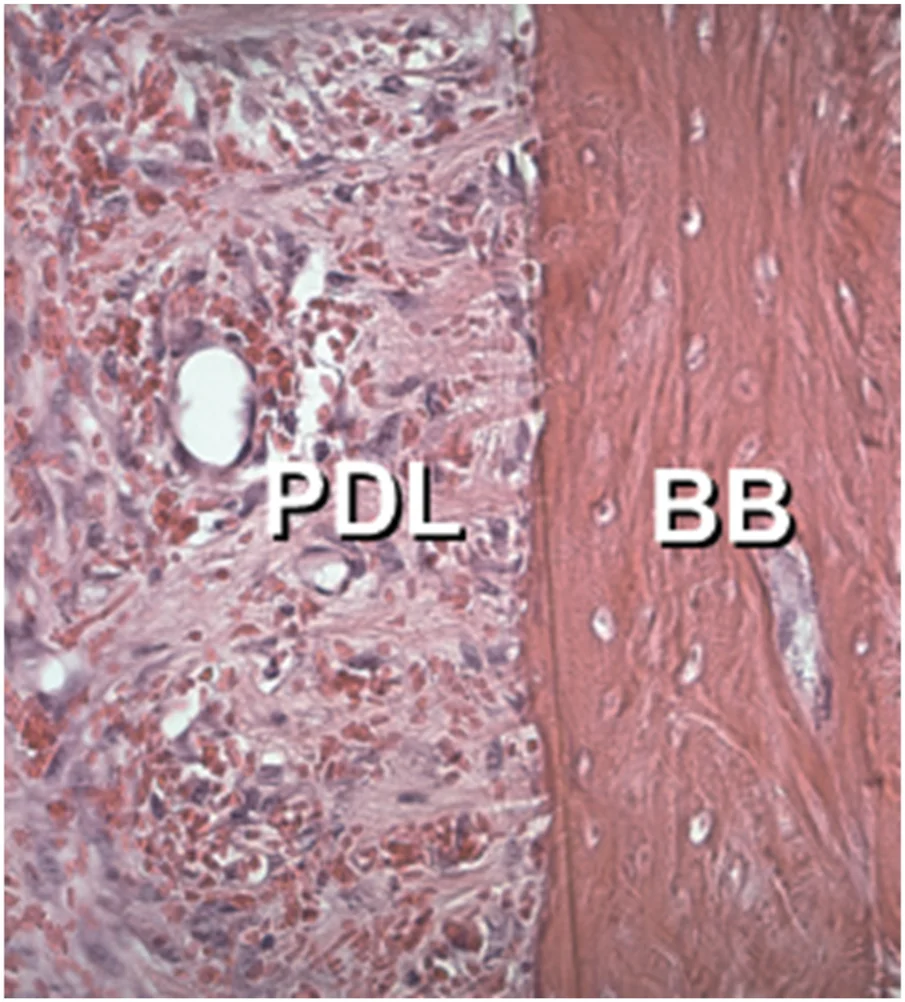
Higher magnification of the outlined area of the healing of the extraction site after 1 week of healing. The bundle bone covered the socket wall. Lateral to the bundle bone, a severed periodontal ligament can be identified. BB, bundle bone; PDL, severed periodontal ligament; original magnification x200. Reproduced with permission from John Wiley and Sons (15) - Copyright © Blackwell Munksgaard 2005.

Araújo and Lindhe (15) studied the hard tissue reactions to tooth extractions. Consequently, after removal of the tooth from the alveolar process, woven bone fills the intra-alveolar part of the extraction site, followed by cortical ridge remodeling, and then turns into bone marrow, resulting in an alveolar defect, partial restoration, loss of bone mainly in the facial aspect of the ridge horizontally, and vertical ridge height (Figure 3). Both bone growth into the socket and the resorption process happens concomitantly and the outcome of the remodeling usually shows as loss of bundle bone (Figure 4), reduction of buccal bone thickness and height. and relocation of the ridge to more palatal/lingual position in histological examination shown in the time periods of 1 week, 2 weeks, 4 weeks, and 8 weeks of healing (Figure 3) (15). About alterations of the height of the bone crest from the 1-week interval, the buccal bone crest location was found to be on average 0.3 ± 0.2 mm coronally to the lingual crest. While at the 2-, 4-, and 8-week intervals, the buccal crest located apically to its lingual counterpart after 2, 4, and 8 weeks of healing were 0.3 ± 0.1, 0.9 ± 0.3, and 1.9 ± 0.2 mm. Thus, the relative reduction of the height of the buccal bone wall between the 1- and 8-week intervals was 2.2 ± 0.2 mm, i.e., about 45 μm/day. The buccal walls thinned at all levels over time. In contrast, the lingual walls underwent slight resorption but remained relatively the same thickness (21).

Tissue alterations after tooth extraction were investigated in beagle dogs to evaluate whether surgical trauma affected the resorption rate. Five 1-year-old beagle dogs were used as subjects to perform extraction on mandibular first and second premolars separated into four groups: treatment group (1): the extraction socket was left with a blood clot; treatment group (2): surgical trauma was performed with a mucopeiosteal flap elevation, crestal incision with two vertical release incisions, and suturing; treatment group (3): after tooth extraction, Bio-Oss was applied into the sockets without any hydration, and a free soft tissue graft was sutured to cover the socket; treatment group (4): surgical trauma was applied in the same manner as in group 2, Bio-Oss was applied into the sockets, and a free soft tissue graft was sutured to cover the socket. Then, polyether impressions were taken at 2 and 4 months after extraction, and the casts were fabricated, scanned, superimposed, and calculated using digital image software. The comparison resulted in the exposure of the buccal bone, which could additionally shrink the alveolar bone by 0.7 mm in volume. This implies that exposure to surgical trauma further affects the resorption process after tooth extraction (22).

Bone healing and soft tissue contours change in dimensions, as shown in several human studies using various methods including clinical, histological, cast models, and radiographic examination. It can be expected that up to half a reduction of the initial ridge will follow single-tooth extraction, and the amount is greater at the buccal side than at the palatal/lingual side, and more in the molar regions, as stated by Pietrovski and Massler (12). Eventually, the socket opening closes by epithelialization of the soft tissue and/or bone regeneration, as seen radiographically (16, 23–26). Soft tissue closure can be clinically observed at approximately 10–20 weeks, while bone fill can be observed radiographically between 3 and 6 months after exodontia. In the first 3 months, most of the dimensional changes occur, and remodeling of the alveolar ridge may continue for up to 1-year after post-extraction. This varies individually in accordance with socket size (large vs. small) and the degree of surgical trauma during the extraction steps (27). A greater loss of bone and drastic dimensional changes in hard and soft tissue after the loss of a tooth would compromise the requirement for reconstruction of the site with dental implants, in which sufficient bone volume and architecture are needed for functional and esthetic prosthetics (2, 16).

Originally, this protocol required a healed socket for implant placement. Implant placement at the time of extraction or immediate implant placement (28) was initially published with the Türbingen implant, a ceramic implant made of Al2O3, evaluated by Professor Wilfried Schulte from the University of Tübingen in Germany in 1978 (29, 30). Because dimensional changes alter the alveolar ridges post-extraction, the delayed timing of implant placement tends not to encourage patients to wait for at least 6 months for the bone to heal completely and becomes less practical in daily situations (31, 32). The protocols for placement and loading are described in the ITI consensus statement of 2018. These options include the following: (a) immediate implant placement on the day of extraction (Type 1), (b) early implant placement after 4–8 weeks of soft tissue healing (Type 2), (c) early implant placement after 12–16 weeks of partial bone healing (Type 3), and (d) late implant placement after complete bone healing for at least 6 months (Type 4) (33). Finally, minimally invasive tooth extraction techniques could benefit and least compromise tooth extraction wounds and osseointegration (34). To minimize surgical trauma and preserve the extraction site for immediate implant placement, the purpose of this review was to demonstrate current and innovative approaches for tooth extraction.

## 3 Minimally invasive extraction techniques and approaches

Minimally invasive extraction techniques and approaches involve additional equipment that helps with cutting, pulling, and changing the fulcrum points and moments to mechanically separate the tooth and alveolar bone from the periodontal ligament, with flap operation changing pulling to pushing the tooth from the apex of the root, or enzymatic chemical degradation of the fiber attachment to decrease the pulling force for tooth extraction to ease the process. All these aim to achieve the same goal of successful tooth removal and minimal surgical trauma for hard and soft tissue wound healing.

### 3.1 Periotomes

With its thin metal blade, a periotome can be used to softly cut the periodontal ligaments or perform a syndesmotomy to separate Sharpey fibers, securing the tooth in the socket. After most of the fibers are cut, the minimal lateral force with the rotating movement can facilitate tooth removal (35).

**Figure 5:**
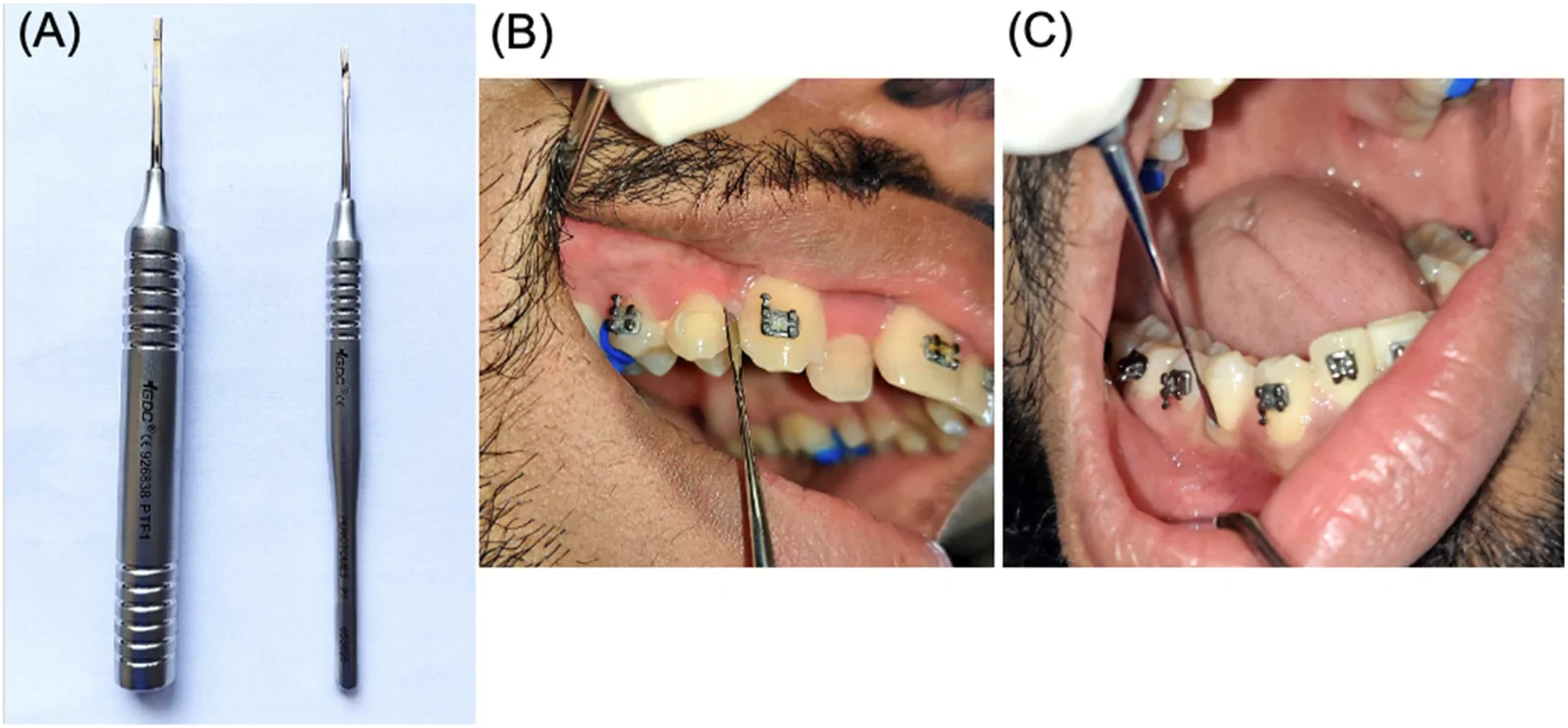
Periotome. **(A)** GDC—flexible periotome (PTF1) and GDC—conventional periotome (PT1), used to detach the PDL by **(B)** Conventional periotome **(C)** Flexible periotome. **(A)** reproduced from “GDC—flexible periotome (PTF1) and GDC—conventional peritome (PT1)”, **(B)** reproduced from “Conventional periotome being used to detach the periodontal ligament wrt 24”, **(C)** reproduced from “Flexible periotome being used to detach the periodontal ligament attachment” by Zoya Rafiqorcid, Kolari Vinayakrishnaorcid and Joyce P. Sequeira, licensed under CC BY 4.0.

A double-blind randomized controlled trial conducted by Sharma et al. (36) compared the use of periotomes to the conventional extraction technique for single-rooted tooth extraction in 100 patients evaluated for postoperative pain at the time intervals of 3rd hour, 6th hour, 24th hour, and 7th day after treatment on a visual analogue scale (VAS) score. Although no postoperative medications were prescribed, the frequency of analgesic consumption and complications were recorded. In the test group, the operation was carried out with the use of the Amron periotome with the blade attachments held in a modified pen grasp inserted into the gingival sulcus at an angle of 20 degrees, severing cervical fibers, further insertion into the PDL space mesially and distally to the root surface, repeating the same motion until reaching two-thirds of the distance toward the root apex. Finally, extraction forceps were applied in the coronal direction with rotational force to extract the tooth (Figure 5). Overall, pain reduction and gingival laceration were significantly reduced in the test group, while mild pain lingering through to the 7th day was significantly more common in the control group. The operation time in the test group was 5.78 min compared to 12.81 min on average in the control group. The frequency and amount of analgesics administered were lower in the test group.

To assess the efficacy of periotome, Contractor et al. (38) compared it with conventional forceps extraction in terms of socket preservation and reduction of postoperative pain. In the periotome group, to loosen the tooth, a periotome was used in a “walking motion”, then forceps were used, followed by minimal rotation and a gentle coronal pulling motion. In the conventional forceps group, conventional root forceps were used with minimal buccolingual and rotational movements followed by a coronal pulling motion to extract the tooth. The visual analog scale (VAS) score and medication use were particularly lower in the periotome group than in the conventional forceps group, and better socket preservation with periotome use was notable. Although the operation time was not different, the difference between both groups might be due to the operator's careful and precise techniques (38). Similarly, another study by Sahu et al. (39) performed a comparative evaluation of the universal periotome and conventional techniques in single-rooted tooth extractions and found that the universal periotome showed superior outcomes, significantly reducing postoperative pain, operative time, gingival lacerations, and analgesic consumption. The minimally invasive periotome technique not only preserves alveolar and gingival architecture but also makes it an effective alternative for single-rooted tooth extractions. These results support the idea that using periotomes aids in minimally invasive extraction, which could benefit postoperative pain control, less surgical trauma, and possibly a faster operation.

Variations in the properties of periotomes have also been investigated for their efficacy in a recent prospective, randomized controlled trial comparing flexible periotomes with regular non-flexible periotomes. Wound healing outcomes, duration of the procedure, and level of gingival laceration were assessed for bilateral tooth extractions on the same jaws in the same patients. The experiment was performed in the same manner as for the conventional periotome group (Figure 5). The use of flexible periotomes resulted in faster operation time at an average of 4.43 min compared to 7.2 min with regular periotomes, and a smaller amount of gingival laceration was achieved. Both groups showed excellent wound healing on the 7th postoperative day, but wound healing with flexible periotomes demonstrated a superior outcome according to Landry's wound healing index assessment (37).

### 3.2 Periotomes and piezotomes

**Figure 6:**
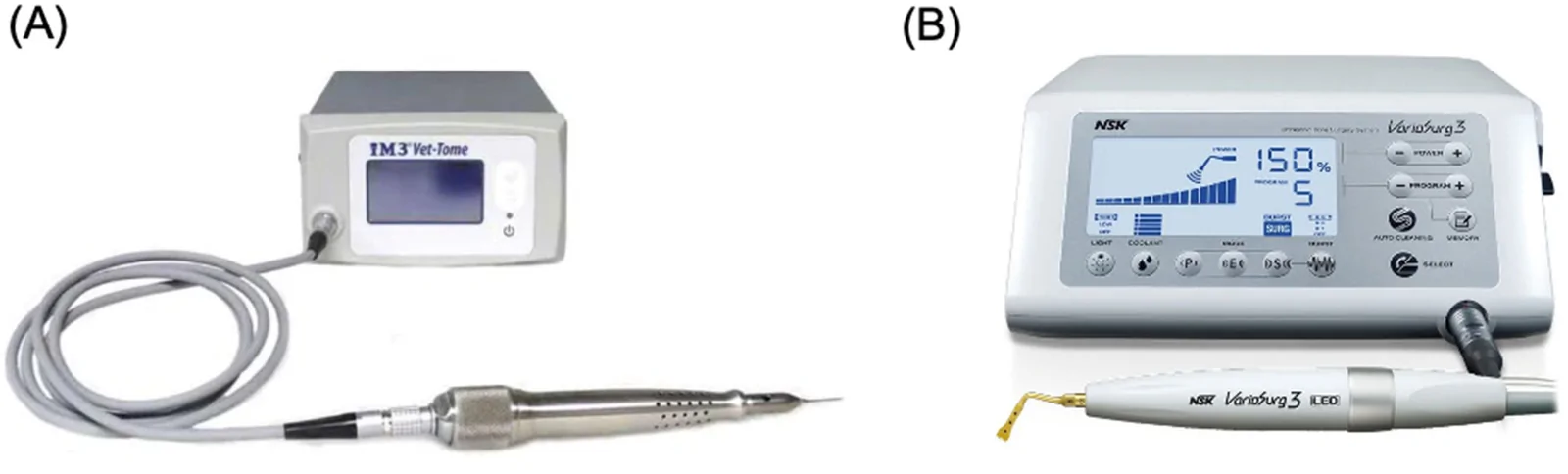
Periotomes and piezotomes. **(A)** Periotome instrument (iM3 Vet-Tome, iM3, Sydney, Australia) and **(B)** piezoelectric device (NSK, Tokyo, Japan).

Newer techniques include the use of periotomes with mechanized automated movement speed, namely powertome periotomes, piezoelectric devices, or piezosurgery, which causes vibration at an ultrasonic frequency (Figure 6). When in combination with periotomes, piezotomes help cut hard and soft tissue more accurately.

An *in vitro* study performed in sheep jaws to compare automated periotomes with conventional periotomes showed comparably better surgical outcomes; however, in the automated group, less surgical trauma as a result of fewer bone fractures was notably demonstrated (40). The use of the powertome periotome was reported in a preliminary study by James Kang, in which extractions utilizing the machine were performed for an average of 4.8 min with great patient satisfaction feedback (41). A series of seven case reports on the use of the powertome periotome in assisting minimally invasive extraction showed how the powertome periotomes work and some of their outcomes in preserving bone housing (42, 43).

Piezoelectric devices convey an ultrasonic oscillating frequency between 29 and 32 kHz, which is suitable for the cutting of hard tissue. With great tactile control and precision, this type of tool can provide favorable wound healing. A comparison was made between conventional extraction and piezosurgery in patients who needed bilateral maxillary or mandibular tooth extractions and found that the ultrasonic tool resulted in a postoperatively better VAS score and less bleeding and swelling from patient self-reports (44).

The effectiveness of periotomes vs. piezotomes in minimally invasive extraction was evaluated in a single-blind randomized controlled trial. On average, the procedure took 4.96 min in the periotome group, whereas it was approximately 3 min longer in the piezotome group, which is significantly slower. For soft tissue trauma, piezotomes performed more gently but were not superior to periotomes. A higher VAS score was reported in the piezotome group, which was statistically significant only during the intraoperative phase. Analgesic use was comparable between the groups. In conclusion, based on this study, the periotomes could be more beneficial than the piezotomes (45).

In accordance with a previous comparison (46), the piezotome performance notably resulted in a higher pain score, longer operation time, and more marginal bone loss immediately postoperatively but was not significantly different from the periotome group at the 6-month postoperative interval from another comparative study that explored the periotomes and piezomes in extractions of previously endodontically treated non-restorable teeth (47). These studies support the use of periotomes as a minimally traumatic approach to piezotomes.

### 3.3 Electric (magnetic) mallet

**Figure 7:**
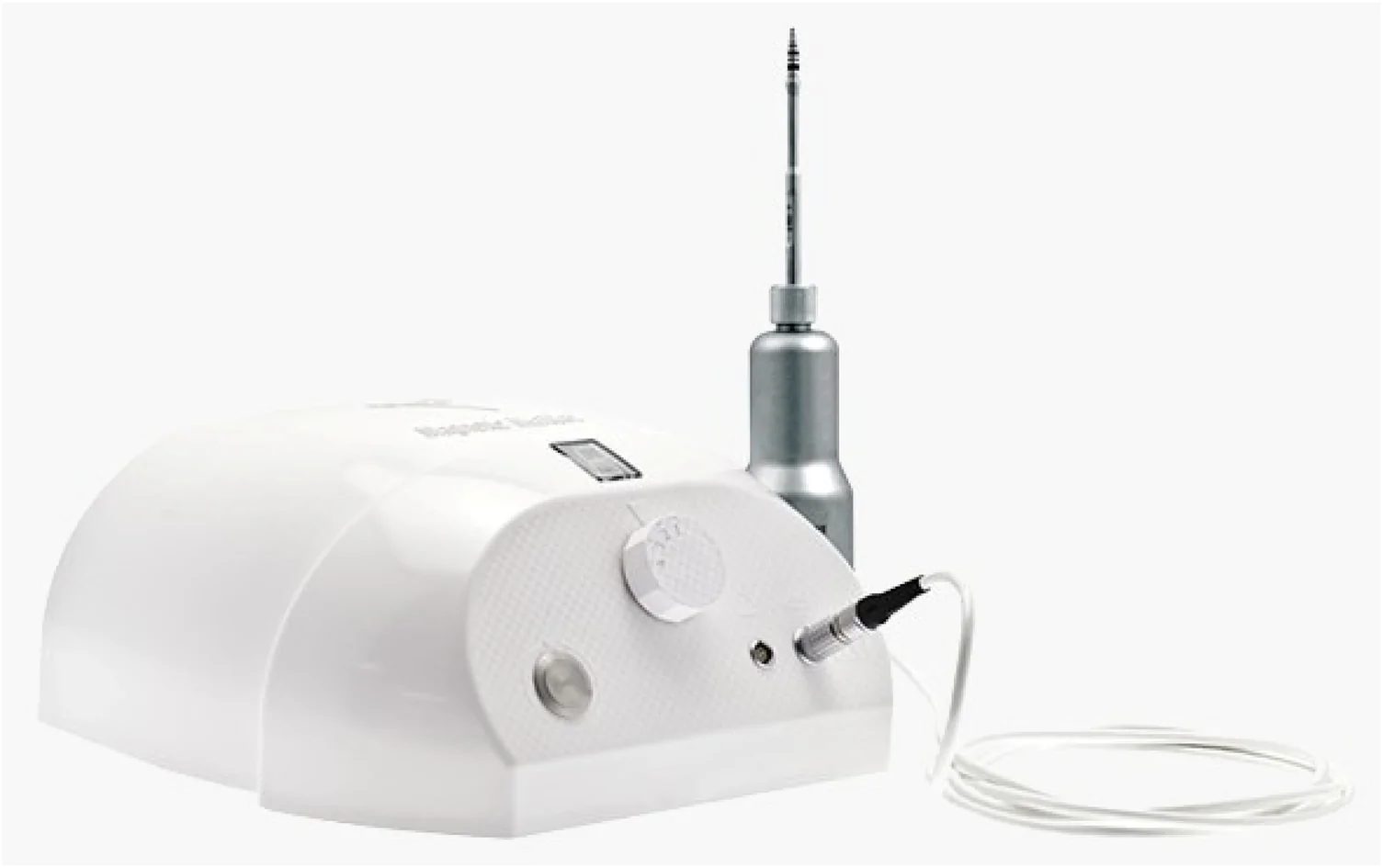
Osseotouch magnetic mallet (Osseotouch, Gallarate, Italy).

The destruction of surrounding structures during tooth extraction can cause bone defects and negative outcomes, affecting treatment with implant placement. Most tools involve the use of the operator's physical force as the extractive force applied to mechanical devices. The magnetic mallet was introduced as a new device to aid tooth extraction with a thin metallic blade containing magnetic pulses and its wave to fluctuate longitudinally apically with a fast force toward the PDL space with minimal hand pressure and to perform syndesmotomy (Figure 7). By focusing the force on the tip of the blade, sweeping axial movements are applied to detach the root from the surrounding bone tissue (48). This requires considerably less effort than conventional periotomes. In a study by Crespi et al. (48), 427 ankylosed teeth were extracted from 156 patients using a Magnetic Mallet, and no loss of cortical bone plate was observed in fresh sockets. The ankylosed teeth were also extracted from one whole piece without the need for more invasive surgical techniques. The electrical mallet reduces potential surgical trauma to the surrounding bones and minimizes damage to the gingival tissues.

One study compared the surgical approaches of the use of an electrical mallet and conventional tooth extraction in maxillary ankylosed teeth, including 66 teeth in total, consisting of 44 maxillary anterior teeth and 22 maxillary premolars distributed unevenly. In the magnetoelectric group, fewer traumatic outcomes resulted in only 2 roots with fractures of the buccal plate. The remaining 38 freshly extracted sites had fully intact buccal bone plates. Eighteen of 28 patients in the conventional tooth extraction group suffered from a buccal alveolar fracture, and the residual ankylotic roots were still attached to the surrounding bone, suggesting more traumatic outcomes, which are unfavorable for socket healing. The need for suturing and postoperative inflammation were also more frequently observed in the conventional tooth extraction group (49).

### 3.4 Sonic instrument for bone surgery

**Figure 8:**
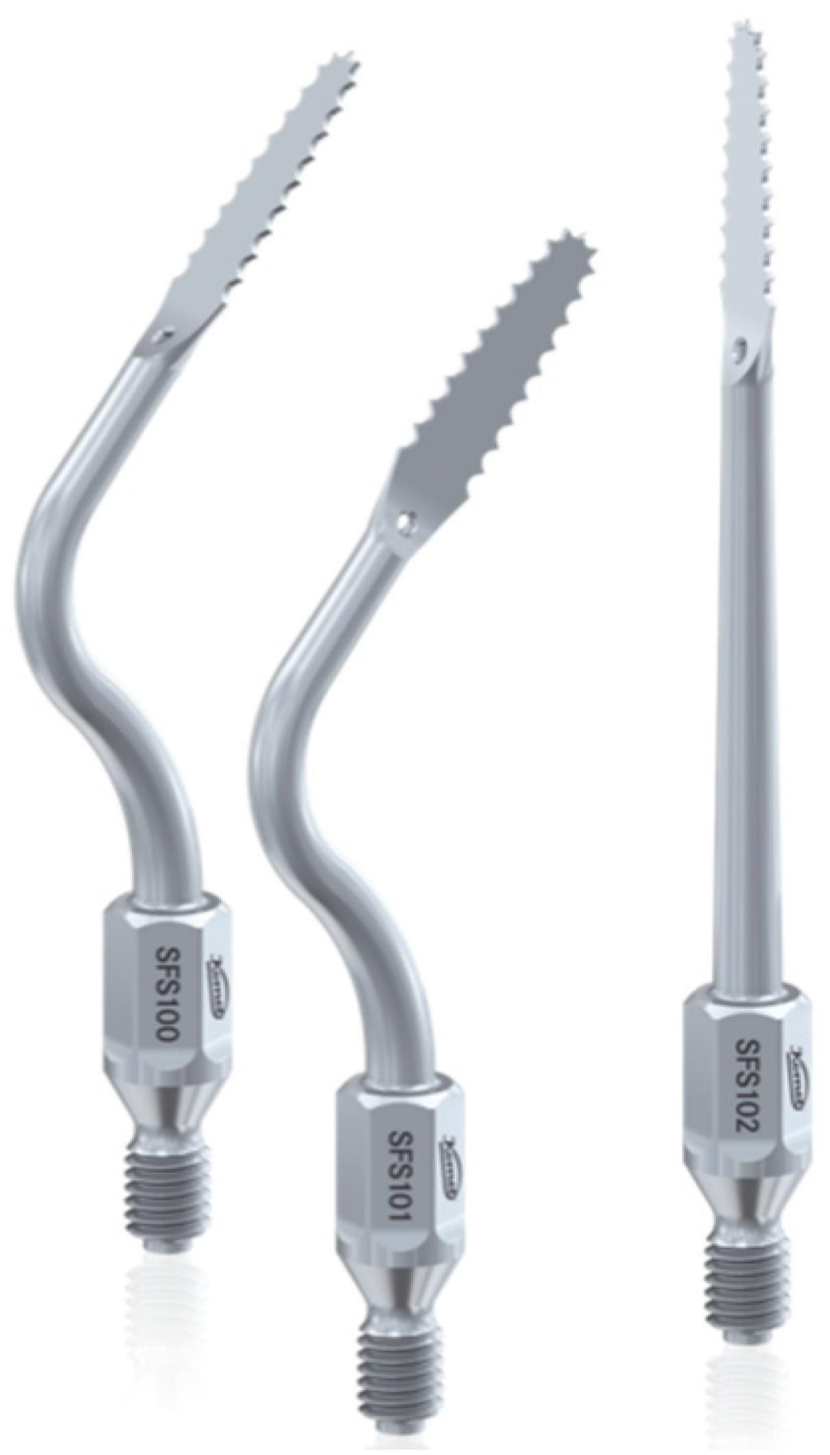
Sonic tips komet dental (Lemgo, Germany) for the extraction of teeth.

Despite the challenges in preserving the completeness of the alveolar bone after tooth extraction, the use of other instruments, such as rotary burs, periotomes, piezoelectric devices, or piezosurgery and vertical extraction devices, are part of these efforts. In a sonic instrument for bone surgery (SIBS), a variety of parts and techniques have been developed to section the teeth and separate the PDL (Figure 8). Originally, these innovative handpieces were meant to aid in finishing marginal preparations for fixed prosthodontics. The device, driven by air pressure, creates vibrations at high frequencies that enable efficient and precise cutting with minimal soft tissue trauma risk. Papadimitriou et al. (50) published a clinical case report presenting a sonic handpiece and its special tips, demonstrating its usage to section tooth roots and expand the PDL space, which opened opportunities to create more effective techniques for minimally invasive extraction. To compare the efficacies of conventional burs, piezoelectric devices, and SIBS, experimental and histological investigations were conducted on porcine mandibles (51). Heat plays a significant role in the traumatology of surgical procedures. It is known that the threshold level for heat-induced cortical bone necrosis is 47 °C for 1 min (52). Conventional burs and SIBS generate less heat at less than 3 °C, whereas piezosurgery can warm up to more than 18 °C even with normal saline coolant irrigation (51). SIBS might be an alternative instrument option that could help dental surgeries with less trauma and greater precision, comparable to piezosurgery but with less heat generation.

### 3.5 Physic forceps

**Figure 9:**
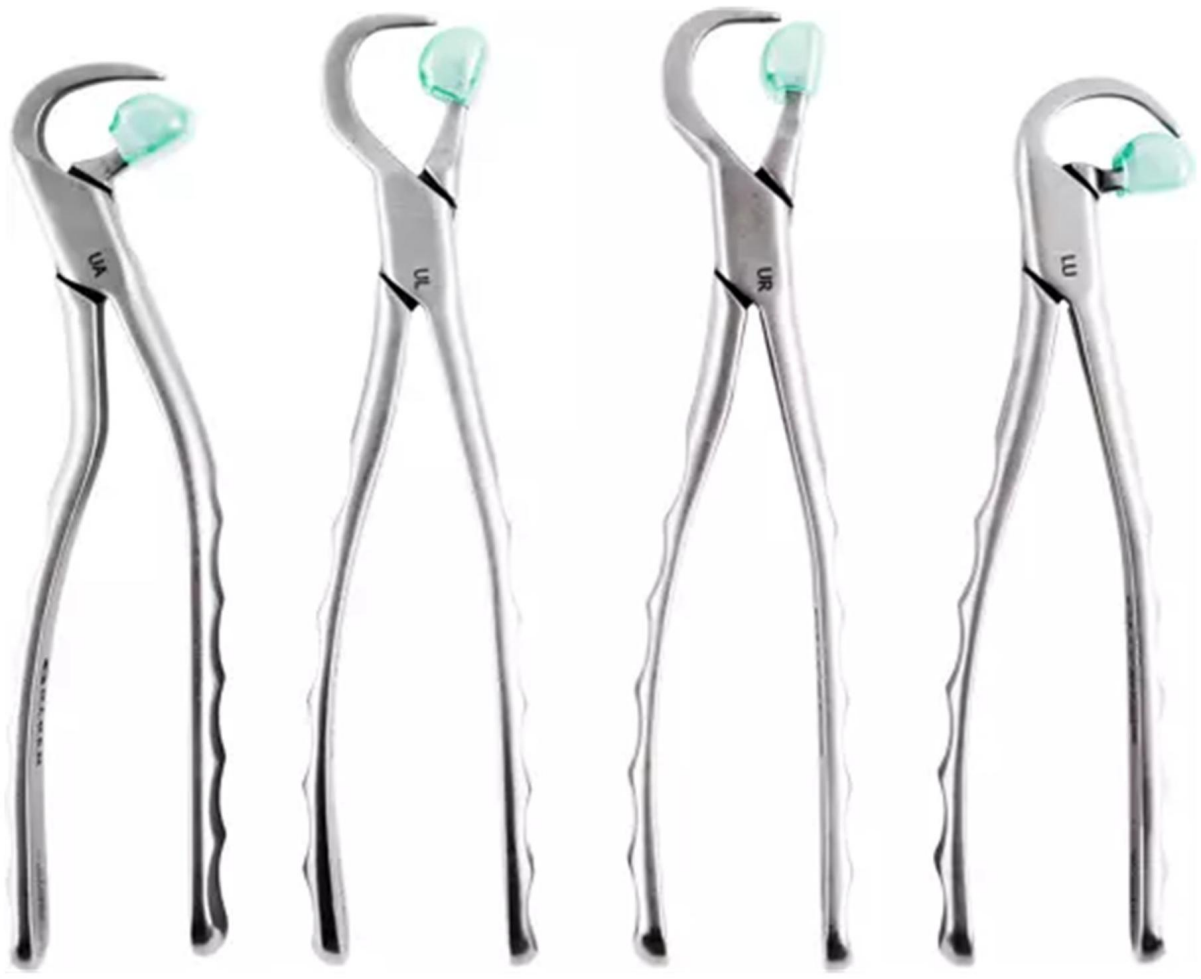
Physics forceps for tooth extraction.

**Figure 10:**
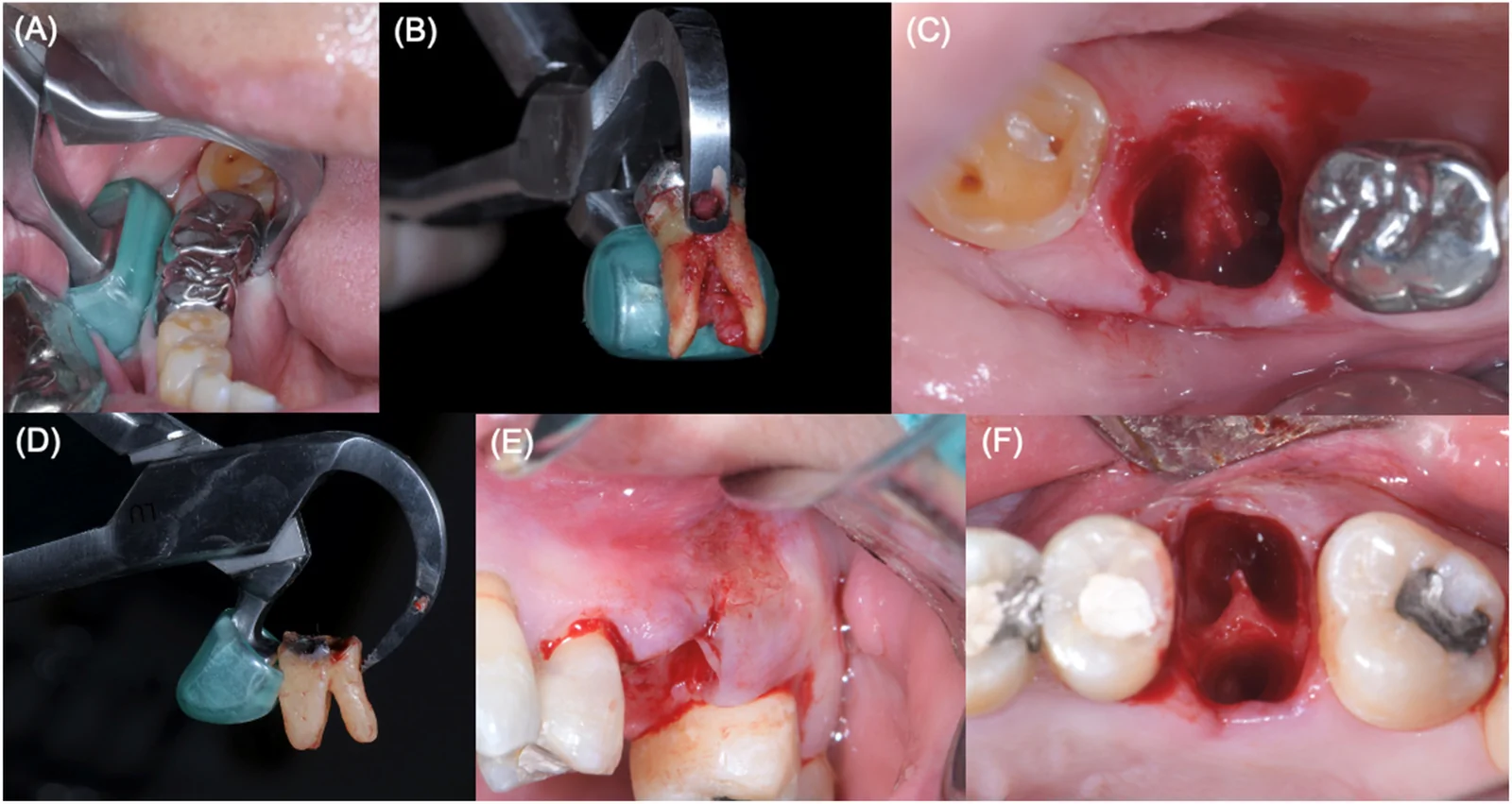
Minimally invasive extraction of the lower molar **(A–C)** and upper molar **(D–F)** using physics forceps. The photos were taken of the patient showing the extraction of lower and upper molars.

This type of dental forceps, developed by Golden Dental Solution based on the first-class lever stress distribution (53), has a beak that engages the tooth at the lingual or palatal surface of the root and utilizes a soft material bumper resting on the buccal side of the socket at the mucogingival junction level, acting as the fulcrum point during tooth extraction, which distributes the extraction force over a large area (43). Several designs offer different extraction sites (53). These are available in the standard series (GMX 100/200), which has a set of four forceps (upper right, upper left, upper anterior, and lower universal) (Figure 9), a molar series (GMX 400), and a pedodontic series (GMX 50). Figure 10 shows the minimally invasive extraction of the lower molar (A–C) and upper molar (D–F) using physical forceps.

The biomechanics of conventional forceps are two first-class levers connected to a hinge where the forces are applied on the long side of the lever, and the beaks are on the short side of the lever, conveyed to the tooth. The hinge acts as a fulcrum but is not involved in tooth removal. The physics forceps involve a single first-class lever; instead of the squeezing force applied to the beak on the tooth, only the force is applied to the lingual side of the tooth root. Torque was applied to the tooth, periodontal ligament, and bone, generating eight times more mechanical advantage owing to the length of the forceps beak (1 cm) and the distance from the handle to the bumper (8 cm) (35).

The application of constant loads to the bone and periodontal ligament is useful for changing the shape of the bony socket and extracting teeth from it. The elastic and ultimate properties of compact bone tissues were described by Donald et al. (44). The bone with a constant applied load undergoes changes into three different stages. Over the first minute, at a constant load of 60 MPa, the bone is strained. This is followed by a higher force application, which causes secondary creep that deforms and expands the socket, allowing the tooth to move out for 1–5 min. The longer the force applied, the greater the deformation of the bone tissues by 10%–20% compared to that in the first minute. Finally, when loads are applied over a longer period, creep fractures of the bone and PDL occur until the tooth is sufficiently loosened to be removed (35). With unidirectional movement as circumferential rotation towards the buccal side, the forceps tear the periodontal ligament off by the applied force, which significantly reduces gingival laceration and buccal cortical plate fracture. The design has a better biomechanical advantage than the lever class for bone expansion from creep and can better distribute stress. The following outcomes utilizing the physics forceps—significant tooth fracture reduction, faster operating time, easier technique, less bleeding, less postoperative infection, less painful postoperatively, and improved healing–were reported in a systematic review with a limited number of current studies and a lack of consistency in the results (46).

To determine the efficacy of physics forceps on the incidence of root fracture and buccal plate maintenance compared to conventional forceps 200 patients were recruited. Crown and root fractures were found more frequently in the conventional forceps group than in the Physics forceps group, while a comparable number of buccal plate fractures were observed in both groups (54). Another comparison in a single-blind, split-mouth clinical trial in 27 patients demonstrated significantly lower postoperative pain scores on the 1st day with Physics forceps; other parameters were similar in both groups (55). Physical forceps showed no significance in the success of extraction, as both physical and conventional forceps faced the same issues of fracture of either root or buccal plate or adherence of buccal plate to tooth, while physical forceps performed with less time consumption and superior VAS score on the 3rd postoperative day, but similar on the 5th and 7th days (56).

Another systematic review and meta-analysis reported that physical forceps performed better than conventional forceps in terms of extraction duration, post-extraction pain, trauma to soft and hard tissues, and complications, with a high risk of bias and low certainty of evidence (57). Owing to the variety of measurement methods and experimental models used in various studies, the methodology could be improved to illustrate whether physics forceps could provide a more minimally invasive extraction approach.

### 3.6 Vertical extraction techniques

An alternative approach has been proposed to aid minimally invasive extraction procedures and prevent bone exposure, which can have destructive consequences for wound healing. An elastic orthodontic band placed at the cervical part of the tooth tends to move, sliding from the larger circumference at the cervical part toward the smaller circumference at the apical part, and loosen the tooth, resulting in gradual extrusion of the tooth. This technique is suitable for a single conical root. Root canal treatment is necessary for vital teeth, followed by tooth sectioning to separate each root in the case of multiple-rooted teeth. This method was applied to 10 patients treated with intravenous bisphosphonate medication for breast cancer, multiple myeloma, and osteoporosis; thus, there was an improved risk of jawbone osteonecrosis. During the application time of 5.8 weeks on average, 13 of the teeth were exfoliated spontaneously, while 2 of them required forceps to remove without the need for suture or antibiotic prescription. No signs of inflammation or bone exposure were observed in any case for up to nine months (58). This study introduces a minimally invasive extraction technique in the absence of surgical trauma to the extraction site using vertical exfoliation in this slow extraction process.

**Figure 11:**
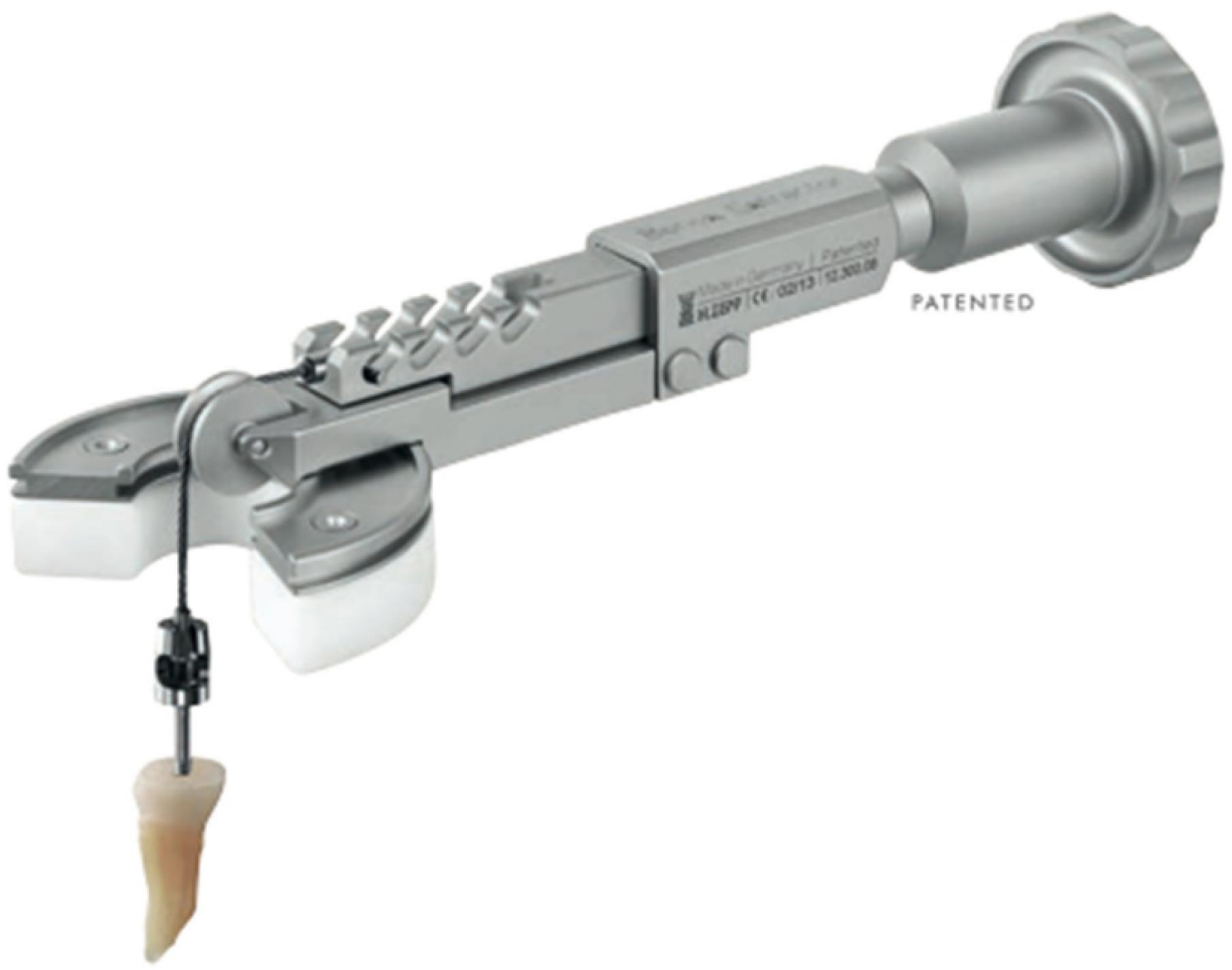
Benex extractor showing an apparatus with the pull rope (Hager & Meisinger GmbH, Neuss, Germany, and Helmut Zepf Medizintechnik, GmbH, Tuttlingen, Germany) for minimally invasive extraction.

Although tooth extraction involves socket expansion using dental forceps, luxators or periotomes can cause bone trauma due to horizontal movement or rotation. Furthermore, teeth that cannot be managed using conventional instruments would require mucoperiosteal flap elevation, often followed by bone removal to enable tooth extraction, which could lead to later bone loss (59). Minimally invasive vertical extraction devices have been developed and are used without the need for flap surgery to reduce trauma to the alveolar bone during tooth extraction (59, 60). By pulling a conical root axially, less trauma could be exerted by vertical extraction systems such as the extractor, Easy X-TRAC, Apex control, and other types of tools that employ similar movements for extraction. When screw devices are attached to the tooth root, an extraction force is applied through the screw and transferred through dentin (61). Each system requires an initial gross caries removal and tooth preparation for the anchor to be screwed into the root canal, after which an extraction force is applied. The Benex extractor (Hager & Meisinger GmbH, Neuss, Germany, and Helmut Zepf Medizintechnik, GmbH, Tuttlingen, Germany) consisted of a Benex extractor, set of diamond burs, set of self-tapping screws, pull strings, and sectional impression trays (62), as shown in Figure 11. When minimal tooth tissue remains, the screw hole in the center of the root is prepared for a self-tapping anchor screw to be attached securely into the root. A pulling string was placed at the head of the anchor. To ensure proper positioning and support for the system, a silicone impression material was placed in the sectional impression tray. The T-bar at the end of the extractor was placed at the notch of the device. After the preparation and insertion of the system, a pulling force was gradually applied by turning the knob at the extractor to extract the root from the alveolar bone (61). This technique is useful for the esthetic anterior region of the maxilla.

The vertical extraction system using the Benex extractor was investigated in a study by Muska et al. (63) to evaluate its applicability, success rates, and limitations. Of the 72 included patients, extraction was planned for 111 teeth. Most of the teeth had retained roots (74%), were single-rooted teeth (87%), and were not previously treated with a root canal (78%). A small fraction (6%) of the samples was included after failure of extraction with conventional forceps. The procedure began after a standard preoperative evaluation and administration of local anesthesia. Initial caries removal, canal preparation, and silicone impression were performed prior to installation of the screw into the prepared canal. The Benex apparatus and pull rope were then inserted, followed by vertical extraction force application. The extracted tooth and socket had minimal surgical trauma and were presented to the tissues. Summing up to an 83% success rate, 19 failures were analyzed, and these occurred in multi-rooted teeth. The main reasons for unsuccessful attempts were lack or loss of retention of the screw, misplacement/misalignment of the screw, fractures of roots due to canal preparation, remaining carious tissues in the root canals, and a variety of root morphologies, such as divergent roots and hypercementosis. The authors suggested that the system could be used for minimally invasive extraction most successfully in single-rooted teeth, and the lack of screw retention could lead to failure of the procedure (63).

Similar outcomes were reported by Hong et al. (59) with a success rate consistent with the findings of Muska (85.4%). A vertical extraction technique was introduced to patients whose teeth were not suitable for conventional methods because of the limited remaining coronal tooth structure or crown fractures during forceps extraction. If the vertical extraction failed, the conventional method or flap surgery was considered appropriate. Specifically, this study reported varied success rates in single-rooted teeth (87.2%) and multi-rooted teeth (69.7%). To analyze failures in this regard, multi-rooted tooth extractions were 2.2 times riskier than single-rooted cases. Root canal-treated roots were 2.1 times more likely to result in failure. Flap surgery was performed in some failed cases, accounting for 5.6% of patients in the vertical extraction cohort. The failed cases in multi-rooted extraction were similar in nature, in that the morphology of the roots and loss of screw retention, especially in the root canal-treated brittle teeth, were possibly due to changes in the root dentin, as discussed by the authors.

A case report using another system for vertical extraction, a neodent dental extractor, was conducted in a 40-year-old male for the extraction of the maxillary left lateral incisor that suffered a horizontal fracture at the marginal gingiva level, which was deemed an unfavorable prognosis for rehabilitation. Before similar tooth preparation protocols for vertical pulling systems were followed, syndesmotomy was carefully performed. With the pin tractor attached to the root, the tooth root was pulled up with the cable rope. The socket was prepared for immediate placement of a Neodent Morse taper implant with an immediate temporary crown (64). In addition to another case report from Brazil employing a Neodent dental extractor for extraction, followed by immediate implant placement with an immediate temporary crown of the maxillary right central incisor in a 40-year-old female patient, excellent performance was achieved with this approach (65). There was also a case report using a different vertical extraction system, the Sapian root removal device system, followed by immediate implant placement in the area of the maxillary second premolar, which showed successful minimally invasive extraction, suggesting another variant of the usable vertical extraction system (66). Minimally invasive vertical extraction associated with immediate implant placement can maintain a harmonious and esthetic outcome of tissue preservation.

### 3.7 Vestibular root extraction technique

Randomized controlled clinical trials comparing different minimally invasive extraction techniques are lacking. There is little evidence from case reports or case series, and few studies have compared them with conventional tooth extraction. Claiming a new minimally invasive extraction technique, vestibular socket therapy for implant placement was performed in 30 patients having a single non-adjacent maxillary anterior tooth missing coronal tooth structure, type II socket (deficient labial plate of bone and intact overlying soft tissues), adequate palatal bone, ≥3 mm apical bone to engage the immediately placed implants, thereby achieving optimum initial torque value (a minimum of 30 Ncm insertion torque) following tooth extraction. The control group underwent incisal extraction, and the test group underwent vestibular root extraction (VRE) followed by conventional forceps. The incisal extraction group started with a sulcular-releasing incision with a microscalpel, followed by interproximal insertion with a periotome between the bone and root surface to cut the PDL into the gingival sulcus along the tooth axis horizontally to the left and right and push further apically until sufficient mobility of the tooth was achieved, which allowed conventional forceps to extract the tooth easily.

The VRE technique was initiated with a 1 cm vestibular access incision–3–4 cm apical to the mucogingival junction of a tooth. The vestibular pouch was incisally elevated to expose the apical root area and gain direct access to the root surface. Slit osteotomy was performed with a long-shanked, small-sized tapered fissure bur at the apical third of the root to separate two-thirds of the coronal part from the remaining apical part. The coronal part was pushed with a straight elevator with axial rotational movement to remove the root incisally, and the remaining apical part was pushed with a Lucas curette. The implant was immediately placed using a prefabricated computer-aided design/surgical guide. The gap was filled with 75% particulate autogenous bone mixed with 25% inorganic bovine mineral bone matrix and covered with a flexible membrane shield. Suturing the vestibular incision and placing a customized PEEK healing abutment to seal the socket were the final steps of the surgical process.

The outcomes of both groups were compared on the day of the final restoration delivery and at 12-month postoperative follow-up using small VOF CBCT STL file superimposition measurements. A statistically significant difference resulted from individual and overall pink esthetic scores of the test group at 12.67 ± 1.59 and 11.40 ± 1.4 [mean difference of 1.27 with 95% CI (0.15, 2.39)] (67).

### 3.8 Enzymatically assisted tooth extraction

Some cases of tooth removal are simple and involve only socket expansion and forceps delivery, while others require a flap operation and alveolar bone removal or odontectomy to separate the tooth root, which could lead to fracture of the root and/or surrounding bone, bleeding and hemorrhage, soft tissue damage, infection and inflammation, paresthesia, or even necrosis of the bone jaw in patients receiving anti-resorptive or antiangiogenic medication. Currently, improvements in technologies, instruments, and techniques to mechanically disrupt the PDL contribute greatly to reducing the risk of complications; however, the reduction of the physical force required for tooth extraction is still an indefinite and unexplored way to benefit tooth extraction.

As the main structure of the PDL that connects the tooth and bundle bone together, collagen can be degraded by collagenases, which are produced by bacteria and mammalian cells. Bacterial collagenase G from *Clostridium histolyticum,* approved for the treatment of Dupuytren's and Peyronie's diseases, burns, and wounds, can digest triple-helix collagen down to short peptides. Tohar et al. (68) transformed recombinant collagenase G (ColG) produced by *E. coli* BL21 with plasmid-containing *Clostridium histolyticum* ColG genes for application in an ex situ model of 6-month-old porcine jaws to disrupt PDL fibers, providing the first evidence for enzymatically assisted tooth extraction. Twelve mandibles of healthy domestic swine with unimpaired teeth, gingival tissues, and alveolar mucosa were included in the experiments on the first and second premolars (PM1 and PM2, respectively, each consisting of two divergent roots: mesial and distal). The jaws were randomly divided into ColG and phosphate-buffered saline (PBS; a vehicle for ColG, which served as the negative control). PM1 and PM2 were exposed up to the alveolar crest by removal of soft tissue and split into mesial and distal parts before being accurately injected with a total of 0.3 mL ColG or PBS per root utilizing a computer-assisted injection system. After injection, the jaws were incubated at room temperature for 16 h to maximize enzymatic activity, and the extraction forces were measured. The force required for tooth extraction was significantly reduced (by up to 50%) after ColG application.

Cellular viability was also tested using Chinese Hamster Ovary (CHO) cells and primary human gingival fibroblasts (hGFs) to evaluate the toxic effects on non-collagen-dependent and collagen-dependent cells by treating them with variable ColG concentrations or PBS. Similar viability was found in CHO cells, showing the safety of ColG on non-collagen-dependent cells, while on the hGFs side, as collagen-dependent cells, the viability was hindered by ColG, as expected. This confirmed that the enzyme was not toxic to the cells.

After the results of the first enzymatically assisted tooth extraction were published, another experiment involving ColG application was performed in an ex situ porcine jaw model. Instead of ColG, the following model applied a computationally designed version of ColG-variant that has superior thermostability at 56.6°C (Thermostability of ColG at 52.9°C), and the ability of the new enzyme was also evaluated; the ColG-variant reduced the force required for extraction by 11% in comparison with ColG (69). It is quite thrilling to learn about this novel, genetic, and epigenetic approach that has great potential to benefit and significantly impact minimally invasive extraction.

### 3.9 Other tooth extraction techniques

The socket-shield technique (SST) for tooth extraction is a partial extraction approach designed to preserve the buccal segment of the root, thereby maintaining the periodontal ligament and bundle bone and minimizing post-extraction ridge resorption (70–72). By intentionally retaining a thin buccal root fragment (2 mm) during implant placement, SST helps preserve soft tissue contours and esthetic outcomes, particularly in the anterior maxilla (73, 74). Histological and clinical studies have demonstrated favorable bone stability and reduced horizontal ridge loss compared to conventional extraction protocols (71, 74). Resorption of the buccal bundle bone is avoided because the biological integrity of the buccal periodontium (bundle bone) remains untouched (75).

Similarly, root sectioning techniques, as emphasized in Glocker's approach (76), help preserve the bone following tooth extraction. This technique involves the strategic division of multi-rooted teeth to allow controlled, atraumatic removal of individual root segments while preserving the surrounding alveolar bone. Techniques such as vertical sectioning, hemisection, and root separation reduce the need for excessive luxation forces and minimize buccal plate damage (77, 78). These methods align with the principles of minimally invasive extraction and can be integrated into socket preservation strategies, including grafting or partial root retention (79).

Together, SST and root-sectioning techniques bridge atraumatic extraction and ridge preservation, offering clinicians effective options for maintaining alveolar architecture and optimizing outcomes for subsequent implant therapies.

## 4 Discussion

Socket preservation is possible at post-extraction sites with thin buccal plates (<2 mm), areas with increased esthetic risk, highly destroyed walls of the post-extraction sockets, multiple extractions, risk of involvement of some anatomical structures (maxillary sinus, mandibular canal, etc.), and delayed implantation (80). Regardless of the technique used for socket preservation, atraumatic tooth extraction is important.

Post-extraction remodeling of hard and soft tissues results in volume reduction and can lead to esthetic challenges with prosthetic restorations, particularly in the anterior maxilla (62). A successful tooth extraction procedure can be achieved using conventional or surgical approaches, although some invade and damage the hard and/or soft tissues of the extraction site, which has detrimental consequences on wound healing. A study (62) compared the results of atraumatic vertical extraction vs. conventional extraction and studied postoperative volume loss and esthetic compromise at the extraction site and adjacent teeth. They found that both groups showed a significant reduction in volume with esthetic impairment of the soft tissue of the adjacent teeth. But other studies have found that the vertical extraction system results in a high success rate for severely destroyed teeth extractions and reduces the need for flap surgery (59, 60).

Alveolar ridge preservation was developed to address the significant reduction in alveolar bone volume after tooth extraction. The primary goal of this approach is to maintain sufficient ridge width and height so that the site remains suitable for dental implants and other prosthetic restoration (81, 82). It shows that post-extraction bone remodeling and resorption can lead to a reduction of nearly 50% of the original bone volume within approximately 12 weeks (10, 18). Therefore, procedures that can slow or minimize this resorptive process are important to achieve better prosthetic rehabilitation outcomes.

Furthermore, alveolar ridge preservation (ARP) techniques suggest that placing graft material into a fresh extraction socket helps stabilize the blood clot during the early stages of healing. The graft also serves as a scaffold that supports bone formation through osteoconduction during the healing phase and is gradually resorbed as new bone tissue develops and replaces it (10, 17). ARP has been found to limit bone resorption and, when used with dental implants, improves prosthodontic and esthetic treatment outcomes (83). In addition, Atieh et al. (84) showed that ARP of periodontally compromised teeth following extraction has short-term positive effects on ridge height and bone volume and minimizes the need for additional augmentation procedures. Furthermore, ARP may help limit dimensional changes in ridge height and width within the first six months after extraction; however, the certainty of this evidence remains low. Therefore, further long-term clinical studies are needed (85).

**Figure 12:**
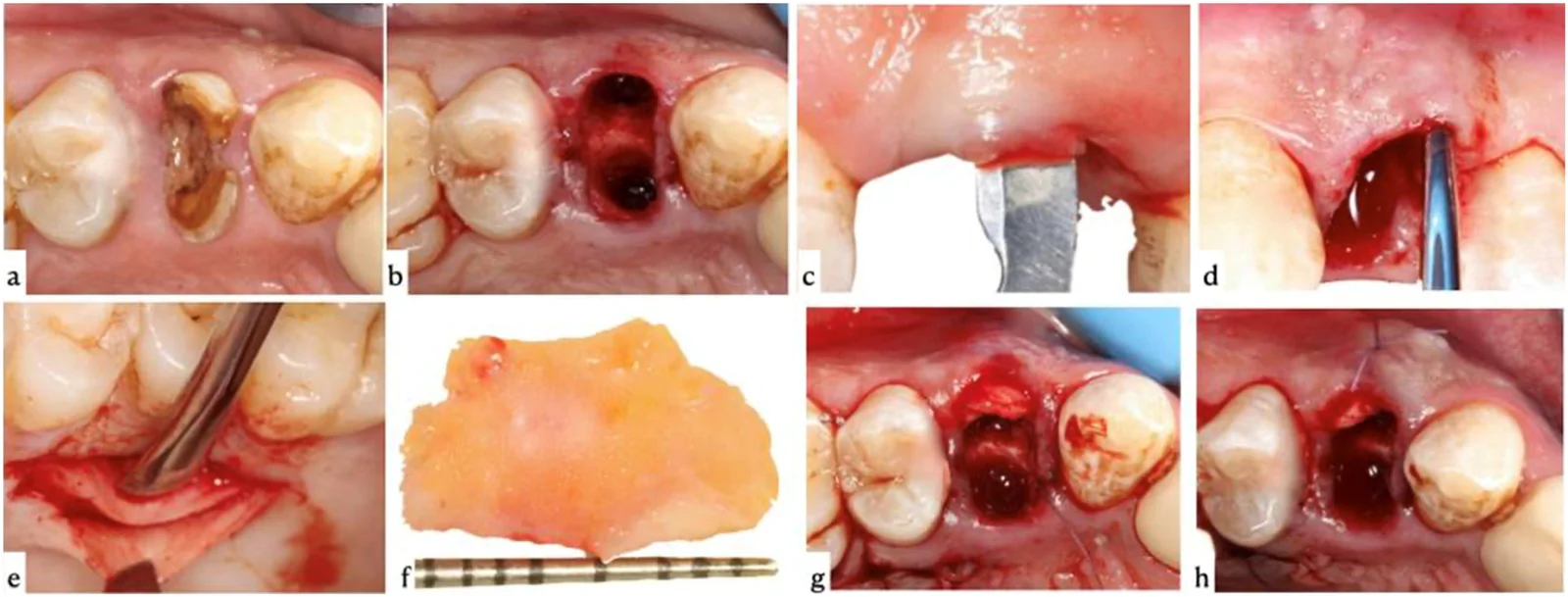
Minimal traumatic extraction using the pouch technique and subepithelial connective tissue grafts following extraction. **(a)** Pretreatment photograph, **(b)** Minimal traumatic extraction, **(c)** split thickness buccal pouch, **(d)** extension of pouch, **(e)** subepithelial connective tissue graft harvesting, **(f)** harvested connective tissue graft, **(g)** subepithelial connective tissue graft placed in the pouch, and **(h)** connective tissue graft secured in desired position by suture. Reproduced from “Overview of minimally-traumatic extraction + SCTG” by Nourhan Gamal, Nesma Shemais, Marwa Al-Nawawy and Noha A. Ghallab, licensed under CC BY 4.0.

Soft tissue augmentation on the buccal side of the extraction socket following tooth extraction can be performed to stabilize the soft tissues and compensate for the buccal concavity that arises after tooth loss (86). Considering this, subepithelial connective tissue grafts (SCTGs) are considered the gold standard in ridge contour augmentation procedures (87, 88). SCTGs provide significant root coverage, clinical attachment, and keratinized tissue gain. Gamal et al. (86) studied the alveolar ridge contour after SCTG of fresh extraction sockets in patients with thin buccal bone vs. minimally traumatic extraction followed by spontaneous healing alone (Figure 12). They found that the SCTG group showed a significant improvement in all outcomes, that is, gain in the buccal soft tissue volumetric change compared to the control group and an increase in gingival thickness and interdental papilla height after 6 months compared to the control group. Hence, the use of buccal SCTG to extract sockets in the anterior maxilla might be considered a predictable approach for preserving the alveolar ridge contour.

However, in addition to the extraction techniques and methods, various other factors can influence wound healing and 3D tissue changes. Studies have shown that increased cigarette consumption, a thin buccal bone wall, and a thin phenotype can affect the postoperative healing process (62, 89). Furthermore, knowledge of the behavior of hard and soft tissues following tooth extraction, especially in the esthetic zone, is required. During healing, it is important to successfully plan the prosthesis for the edentulous space (62). Innovative atraumatic approaches are yet to be explored; furthermore, surgical trauma should be minimized to favor the healing process and implant placement, and this would rely greatly on the intact native structures of the alveolar bone socket to offer the most initial torque value.

Among minimally invasive extraction techniques, piezotome-assisted extraction appears to be associated with the least buccal bone loss. This is largely attributed to its micrometric, selective cutting action that preserves the cortical plates and minimizes mechanical stress on the alveolar housing (90, 91). In contrast, the magnetic (electric) mallet relies on controlled percussive forces to luxate teeth, which, although effective, may transmit stress to the buccal plate and increase the risk of microfractures or remodeling, particularly in thin bones. Sonic instruments facilitate periodontal ligament disruption with reduced manual force; however, their efficiency is operator-dependent and may result in localized trauma if excessive pressure or improper angulation is applied. Hence, piezotomes demonstrate superior precision and soft tissue-bone selectivity, making them more favorable for minimizing buccal plate resorption, especially in esthetically critical or anatomically delicate regions (15).

There are limitations to the tools used for minimally invasive extractions. Contraindications for piezotomes include patients with uncontrolled systemic conditions (e.g., poorly controlled diabetes or bleeding disorders) where prolonged surgical time is undesirable; severe infection or acute abscess; very dense cortical bone requiring rapid bone removal; patients with cardiac pacemakers or electronic implants; and limited access areas where bulky tips cannot be positioned properly. Similarly, contraindications for electric (magnetic) mallets include patients with neurological disorders (e.g., epilepsy) due to percussive impulses, recent implant placement or grafting in adjacent sites, inner ear disorders or a history of vertigo (risk of vestibular disturbance), severely ankylosed teeth, patients with cardiac pacemakers, and psychologically anxious patients intolerant to percussive sensations. Finally, contraindications for sonic instruments include severely curved, divergent, or hyper-cementosed roots (risk of root fracture); advanced periodontal disease; thin cortical plates at high fracture risk; limited mouth opening restricting instrument angulation; and acute infection with excessive mobility. Nevertheless, none of these tools have absolute contraindications; rather, their use depends on case selection, operator skill, and anatomical considerations. Conventional surgical extraction may be preferred when access is limited, the bone is extremely dense, and patient-related factors increase procedural risk.

In addition, some limitations of the review include the selection and inclusion of articles, as some articles may have been missed. In addition, we attempted to include most of the clinical aspects of socket preservation and atraumatic extraction. However, some information may have been missing. Finally, some atraumatic extraction techniques have been well-established, whereas others have only recently been introduced. Therefore, further evidence is necessary to support clinical judgment and applications.

## 5 Conclusion

Tooth extraction leads to structural changes in the quality of the alveolar socket, especially in the facial or buccal bone, which depends on the tooth for biological maintenance. Alveolar bone socket modeling and remodeling occur during the wound healing process of hard tissue, resulting in bone resorption that compromises implant placement. Minimally invasive tooth extraction techniques aim to successfully extract hopeless teeth, minimize the surgical trauma that can have detrimental consequences, and maintain an intact extraction site that favors immediate implant placement. Some atraumatic extraction techniques are well-established, whereas others have only recently been introduced. Therefore, further evidence is necessary to support clinical judgment and applications. Becoming acquainted with these systems and instruments could potentially assist dental practitioners in selecting appropriate tools and enhancing the quality of patient treatment.

## Data Availability

All data generated or analyzed during this study are included in the article. Further inquiries can be directed to Suphachai Suphangul, kungomfs@gmail.com.
